# Prevalence and Distribution of the Middle Mesial Canal in Mandibular First Molar Teeth of the Kerman Population: A CBCT Study

**DOI:** 10.1155/2020/8851984

**Published:** 2020-10-31

**Authors:** Maryam Kuzekanani, Laurence J. Walsh, Mousa Amiri

**Affiliations:** ^1^Endodontology Research Center, Kerman Dental School, Kerman University of Medical Sciences, Kerman, Iran; ^2^The University of Queensland School of Dentistry, Brisbane, Australia; ^3^Private Practice, Iran

## Abstract

**Purpose:**

Missed additional canals are one of the most important reasons for RCT failures in molar teeth. This study aimed to determine the prevalence and distribution of middle mesial canals in mandibular first molars of the Kerman population.

**Materials and Methods:**

A retrospective study was performed on de-identified cone beam image sets from 3 private radiology centers in Kerman. A total of 100 mandibular first molars from 62 patients (mean age 32 years) were included. Information regarding the patient's age and gender, the location of teeth, and the presence or absence of a MMC and also a second distal canal in MMC cases was recorded in an Excel table. Data were analyzed using SPSS software (IBM-USA).

**Results:**

The overall prevalence of MMCs in the mandibular first molars was 8.1% (10.0% in females and 6.3% in males). More cases of MMCs were seen on the right side (12.2%) than the left side (3.4%). One case of bilateral MMCs combined with bilateral second distal canals in the mandibular first molars was seen in a 26-year-old female. A further case of bilateral MMCs was found in a 32-year-old male but with single-canal distal roots on both sides.

**Conclusion:**

The overall prevalence of MMCs in the Kerman population (8.1%) is at the lower end of the reported range of the international literature (0.26% to 53.8%). In the cohort examined in this study, mid mesial canals were more prevalent in females and on the right side. There was no definite relationship between MMCs and second distal canals in the mandibular first molar teeth in the same subject. MMCs may be unilateral or bilateral. Careful exploration of the pulpal floor between canal orifices is essential to prevent missing the MMC, as this would cause undesirable clinical outcomes.

## 1. Background

Mandibular permanent molars are the most common teeth requiring endodontic treatment in dental practice. They play a key role in mastication, so maintaining them is important for effective chewing. The mesial roots of mandibular molars normally have a mesiobuccal (MB) canal and a mesiolingual (ML) canal, whereas their distal roots usually have one centrally placed canal. Between the two canals in the mesial root, there is a narrow connection known as the isthmus, which contains pulp tissue. Within the isthmus area, anatomical variations occur, including the presence of a further canal, the middle mesial canal (MMC), while in the distal root, a second canal can occasionally be found [1].

Barker et al. [2] were the first authors who reported the presence of a third independent canal in the mesial roots of mandibular molars. Later, Martinez-Berna and Badanelli reported a middle canal in the distal root of mandibular first molars [3]. The diameter of a MMC is generally less than that of the two main canals in the mesial root. The mean diameter of the MMC orifice has been reported to be 0.16 mm, three times less than the diameter of the main mesial canal orifices, which is 0.5 mm. The MMC also has a much lower overall volume, 0.20 ± 0.10 mm^3^, in comparison with the MB and ML canals (0.75 ± 0.20 and 0.88 ± 0.19 mm^3^, respectively) [4]. Overzealous preparation of the MMC may cause perforations. Some authors have reported that the MMC is located equidistant between the MB and ML canals, while others report it to be positioned closer to one of the two main canals [5–8].

Pomeranz et al. have classified the cross-sectional morphology of the MMC into three types: independent, confluent, and fin. In the first type, the canal runs independently from the orifice to the apex of the root. In the confluent type, the MMC canal joins either the MB or ML canal before the apex, while in the fin type, an isthmus may be present between the MMC and the MB or ML canal at any point along its length [9].

Thorough debridement of the root canal system is essential for achieving a successful outcome of root canal treatment. Failure to detect the presence of a MMC during root canal treatment of mandibular first molar teeth could allow remnants of pulp tissue and bacterial biofilms to be left behind in this canal, prolonging periapical inflammation and causing failure of the overall treatment. This is particularly likely when the MMC is of the independent type according to the Pomeranz classification, as it constitutes a “missed noninstrumented canal.”

Knowledge of the prevalence and distribution of the MMC in mandibular molar teeth among different populations is important. A number of studies have reported that the variations in root canal morphology and anatomy of molar teeth may be due to racial and ethnic factors, making these anatomical variations occur at different rates in populations living in different parts of the world [10]. In a particular geographic region, similarities in anatomical variations of teeth are more common, since these have a genetic basis and are inherited [11, 12]. Past work has examined the occurrence of MMC in some populations, but the situation in Iran has not been examined previously. This study aims to determine the prevalence and distribution of middle mesial canals in the population of Kerman, a city in the south east of the Islamic Republic of Iran. A particular point of interest was whether the prevalence in females was the same as the males, with the null hypothesis being that there was no difference.

## 2. Methods

Following the approval of the institutional research ethics committee (Approval # Ir.KMU.REC.1394.386), a retrospective analysis was undertaken on existing cone beam data sets that had been coded and de-identified. The cone beam images had been provided at three private radiology centers in Kerman. The cone beam data sets included 100 permanent mandibular first molar teeth in 62 patients (32 males and 30 females). While assessing available data sets, cases, in which the permanent mandibular first molar teeth were unerupted, had incomplete root formation or root resorption, or had coronal caries, were excluded from the study. Consent letters from the patients for using their radiographic data in this study were obtained by the radiology centers. The cone beam scans had been taken during 2017 to 2019 using a ProMax 3D unit (Planmeca, Helsinki, Finland), with patients in the closed-mouth position with maximum intercuspation. The imaging parameters were as follows: field of view 60 × 60 × 50 mm; voxel size 0.12 mm; and exposure time 12 s. The cone beam data sets were assessed by a dentomaxillofacial radiologist, an endodontist, and a further investigator, using Romexis® digital imaging software, version 2.9.2 (Planmeca, Helsinki, Finland) to examine axial and coronal slices (thickness = 0.05 mm).

Details of the patient's age and gender, the presence and location of MMCs, and also the existence of a second distal canal in positive MMC cases were recorded and analyzed using SPSS software (IBM, USA) [13–16].

## 3. Results

The average age across the cohort of 62 patients was 32 years, and the cohort contained similar numbers of males (*N* = 32) and females (*N* = 30) (Table 1). A total of 5 patients had at least one MMC. The overall prevalence of a MMC in mandibular first molars was 8.1% (10.0% in females and 6.3% in males). The overall prevalence of MMC was higher on the right side (5/41 scans, 12.2%) than the left side (2/59 scans, 3.4%).

**Figure 1:**
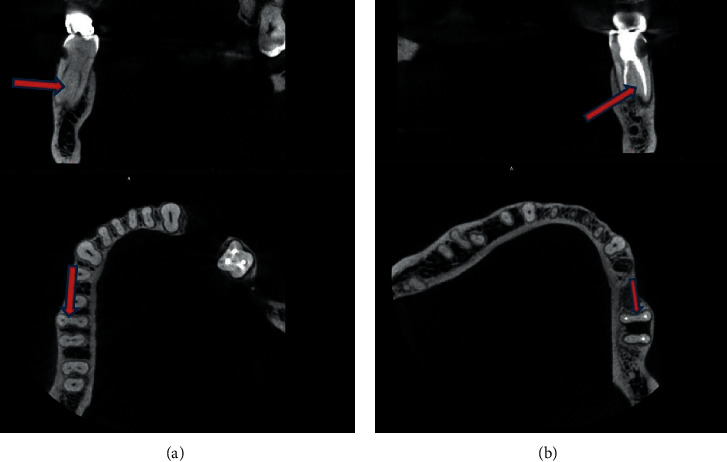
Case 1: a 26-year-old female with bilateral middle mesial canals in the mandibular first molars. The distal roots of the same teeth have two canals.

**Figure 2:**
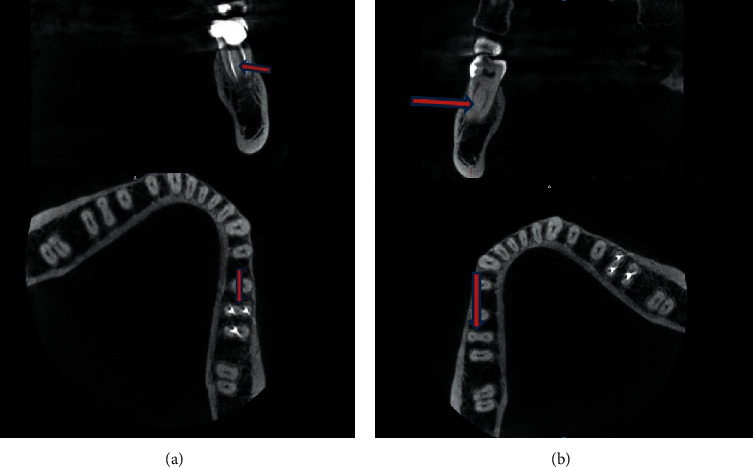
Case 2: a 32-year-old male with bilateral middle mesial canals in the mandibular first molars, showing just one canal in the distal roots.

Two of 5 cases of MMCs were bilateral. In the first case (Figure 1), a 26-year-old female had bilateral middle mesial canals, as well as bilateral second distal canals in her mandibular first molars. In the second case (Figure 2), a 32-year-old male had bilateral middle mesial canals, with single-canal distal roots in the mandibular first molars on both sides.

**Figure 3:**
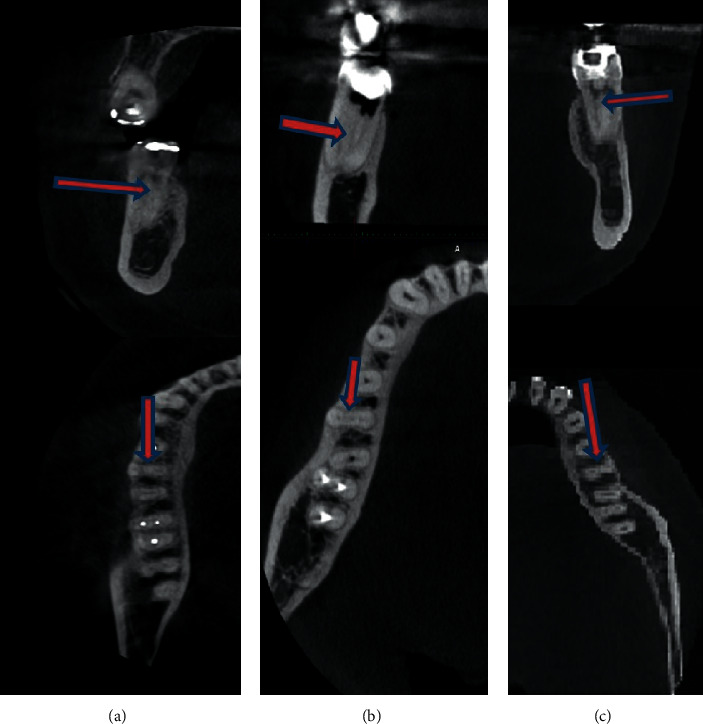
Cases 3 to 5 of mid mesial canals. (a) 42-year-old female. (b) 30-year-old male. (c) 28-year-old female.

Three other examples of MMCs are shown in Figure 3. All were unilateral, and the distal roots had only one canal.

## 4. Discussion

The prevalence of morphological variations in certain types of the teeth is affected by racial and ethnic factors. This explains why morphological features of mandibular molar teeth, including the prevalence of MMC in the mesial root of these molar teeth, occur at different rates in different parts of the world. Such variations could also result from differences in the sensitivity of the methodologies used to detect these anatomical features [1, 13, 17]. The present study used cone beam imaging, which is highly informative for detecting additional root canals in teeth. It has been reported that the accuracy of cone beam imaging with a 60 × 60 mm field of view and a voxel size of 0.125 for detecting a second MB canal in maxillary molar teeth is 96% [16]. The cone beam scans used in the current study had been provided by a field of view of 60 × 60 × 50 mm, and a voxel size of 0.12 mm. This allowed a detailed view of the anatomy of the molar roots.

Other than the cone beam imaging, other methods have been used to look for the presence of MMC in mandibular molar teeth, including troughing the floor of the pulp chamber to locate additional root canal orifices, while using an endoscope or a dental operating microscope [7, 8]. Azim and colleagues in a clinical study involving 91 mandibular molars reported that 42 of these (46.2%) had negotiable middle mesial canals. Six were located after conventional access cavity preparation, while 36 were located after troughing between the MB and ML canals. The authors concluded that troughing helped them locate the middle mesial canal [18]. Troughing that is performed incorrectly (in the wrong direction or too deep) while attempting to locate the orifice of a middle mesial canal could, however, cause iatrogenic mishaps such as perforations and could also weaken the root.

A recent study reported a low prevalence of MMCs in the mesial roots of mandibular first molars in a Chinese population but, at the same time, a high prevalence of isthmuses. Based on this, the authors recommended that cone beam imaging would be valuable for differentiating between isthmuses and middle mesial canals [19]. In line with this, the present study shows the value of using cone beam imaging, as it was possible from horizontal slices of the mesial roots to both detect the presence of middle mesial canal and track the path of this canal to the point where it ended, either at the apex or by fusing with another canal. Having such information from cone beam images in hand before commencing root canal treatment may prevent unnecessary removal of tooth structure with its associated risks of iatrogenic mishaps. Cone beam imaging can be useful for differentiating between a true middle mesial canal and an isthmus.

Using cone beam imaging, Tahmasbi and colleagues [13] examined 122 extracted mandibular molar teeth and found that 20 (16.4%) had true middle mesial canals, with an overall prevalence of 26% in mandibular first molars, and 8% in second molars, with no difference between genders. In their study, female patients were more numerous than males (66% vs. 34%), and the mean age of the patients was 45 years. In contrast, in the current study, the number of female and male patients was almost equal, the average age was 32 years, and the overall prevalence was 8.1%. Differences in age and gender of the study cohort could influence the results obtained. Additional root canals are easier to detect in younger patients because they have less physiologic or pathologic pulp calcifications than older patients [1, 8, 18].

In the present study, a case of bilateral MMCs was found in a female who also had second distal canals in the mandibular first molars, giving 5 canals in total. Others have reported cases of mandibular first molars with six separate canals [20].

Finding both bilateral and unilateral MMC cases in this study is in agreement with another CBCT study on root canal anatomy and morphology of the molar teeth that has been reported; the symmetry in root canal anatomy and morphology of molar teeth is expected to be only in 70% of cases [21].

The present finding of more MMCs in females than males is consistent with other studies that have found significant statistical differences in root canal morphology of certain teeth according to gender [22, 23]. Moreover, a recent systematic review and meta-analysis demonstrated that both gender and the geographic origin of patients can influence the expression of particular morphological features [24].

The root canal anatomy of permanent first molar teeth is an important topic because of the high frequency of endodontic treatment on this tooth in dental practice. As the use of dental operating microscopes, endoscopes [8, 17, 25], and cone beam imaging [26–28] has become more widespread, the detection and treatment of teeth with MMC have been improved, raising the quality of clinical practice.

With an aging population in many parts of the world and more molar teeth being retained until an advanced age, mandibular molar root canal treatment is likely to remain as a major part of clinical dentistry in the future. As the molar teeth are essential for mastication of dense fibrous foods, their preservation is an important factor for maintaining oral health, and for ensuring that patients can choose a wide range of nutritious foods, in line with expectations for a healthy diet. Mandibular molar teeth can be affected by dental caries on the crown and the root surfaces, and also by periodontitis. Both these common conditions may endanger the overall health of the pulp. With loss of periodontal attachment, involvement of the dental pulp can occur through lateral canals once the mandibular molar teeth have furcation involvement. Missing a MMC during root canal treatment could also lead to persisting periapical inflammation. Therefore, having an understanding of how often the MMC occurs reminds clinicians of the importance of this issue when they are commencing root canal treatment on mandibular molar teeth.

Likewise, combined endodontic-periodontal lesions can occur and compromise the longevity of the mandibular first molar as a strategic tooth, with potential implications as a site of persisting infection. Growing awareness of oral health and systemic health interactions [29–31] makes it important to ensure that the patient has no persistent sites of infection and inflammation, and that they are maintaining a healthy dentition throughout their life.

## 5. Conclusion

The overall occurrence of the middle mesial canal in Kerman population (8.1%) is at the lower end of the reported range in the international literature, which is 0.26% to 53.8%. This anatomical variation was more prevalent in females and on the right side in the southeastern Iranian population cohort examined. There was no definite relationship between MMCs and second distal canals in the mandibular first molars in the same subject. MMCs may be unilateral or bilateral. Careful exploration of the region between canal orifices is essential to prevent missing the MMC, as this would cause undesirable clinical outcomes.

## Figures and Tables

**Table 1:** Demographics of the whole cohort and subjects with MMC.

	Whole cohort	Subgroup with middle mesial canals detected
Number of patients	*N* = 62	*N* = 5 (8.1%)
Gender	32 M	2 M (6.3%)
	30 F	3 F (10.0%)
Mean age in years	Males: 32.0 yr (SD 12.1)	31.0 years
	Females: 32.4 yr (SD 11.8)	32.0 years
Location	Right side scans: *N* = 41	*N* = 5 (12.2%)
	Left side scans: *N* = 59	*N* = 2 (3.4%)
		2 of 5 cases were bilateral

## Data Availability

The data used to support the findings of this study are available from the corresponding author upon request.

## Abbreviations

RCT: Root canal therapy
MMC: Middle (mid) mesial canal
MB: Mesiobuccal
DB: Distobuccal.

## References

[1] Hargreaves K. M., Berman L. (2016). *Pathways of the Pulp*.

[2] Barker B. C. W., Lockett B. C., Parsons K. C. (1969). The demonstration of root canal anatomy. *Australian Dental Journal*.

[3] Martínez-Berná A., Badanelli P. (1985). Mandibular first molars with six root canals. *Journal of Endodontics*.

[4] Versiani M. A., Ordinola-Zapata R., Keleş A. (2016). Middle mesial canals in mandibular first molars: a micro-CT study in different populations. *Archives of Oral Biology*.

[5] Sherwani O. A., Kumar A., Tewari R. K., Mishra S. K., Andrabi S. M., Alam S. (2016). Frequency of middle mesial canals in mandibular first molars in North Indian population-an in vivo study. *Saudi Endodontic Journal*.

[6] Nosrat A., Deschenes R. J., Tordik P. A., Hicks M. L., Fouad A. F. (2015). Middle mesial canals in mandibular molars: incidence and related factors. *Journal of Endodontics*.

[7] De toubes K. M., Côrtes M. I., Valadares M. A. (2012). Comparative analysis of accessory mesial canal identification in mandibular first molars by using four different diagnostic methods. *Journal of Endodontics*.

[8] Karapinar-Kazandag M., Basrani B. R., Friedman S. (2010). The operating microscope enhances detection and negotiation of accessory mesial canals in mandibular molars. *Journal of Endodontics*.

[9] Pomeranz H. H., Eidelman D. L., Goldberg M. G. (1981). Treatment considerations of the middle mesial canal of mandibular first and second molars. *Journal of Endodontics*.

[10] Sert S., Aslanalp V., Tanalp J. (2004). Investigation of the root canal configurations of mandibular permanent teeth in the Turkish population. *International Endodontic Journal*.

[11] Sperber G. H., Moreau J. L. (1998). Study of the number of roots and canals in Senegalese first permanent mandibular molars. *International Endodontic Journal*.

[12] Vertucci F. J. (1984). Root canal anatomy of the human permanent teeth. *Oral Surgery, Oral Medicine, Oral Pathology*.

[13] Tahmasbi M., Jalali P., Nair M. K., Barghan S., Nair U. P. (2017). Prevalence of middle mesial canals and isthmi in the mesial root of mandibular molars: an in vivo cone-beam computed tomographic study. *Journal of Endodontics*.

[14] Villas-Bôas M. H., Bernardineli N., Cavenago B. C. (2011). Micro-computed tomography study of the internal anatomy of mesial root canals of mandibular molars. *Journal of Endodontics*.

[15] Kim S.-Y., Kim B. S., Woo J., Kim Y. (2013). Morphology of mandibular first molars analyzed by cone-beam computed tomography in a Korean population: variations in the number of roots and canals. *Journal of Endodontics*.

[16] Mirmohammadi H., Mahdi L., Partovi P., Khademi A., Shemesh H., Hassan B. (2015). Accuracy of cone-beam computed tomography in the detection of a second mesiobuccal root canal in endodontically treated teeth: an ex vivo study. *Journal of Endodontics*.

[17] de Carvalho M. C., Zuolo M. L. (2000). Orifice locating with a microscope. *Journal of Endodontics*.

[18] Azim A. A., Deutsch A. S., Solomon C. S. (2015). Prevalence of middle mesial canals in mandibular molars after guided troughing under high magnification: an in vivo investigation. *Journal of Endodontics*.

[19] Xu S., Dao J., Liu Z. (2020). Cone-beam computed tomography investigation of middle mesial canals and isthmuses in mandibular first molars in a Chinese population. *BMC Oral Health*.

[20] Hashem A. A. R., Ahmed H. M. A. (2016). Endodontic management of a mandibular first molar with an unusual canal morphology: case report. *European Endodontic Journal*.

[21] Plotino G., Tocci N. M., Grande L. (2013). Symmetry of root and root canal morphology of maxillary and mandibular molars in a white population: a cone-beam computed tomography study in vivo. *Journal of Endodontics*.

[22] Kazemipoor M., Hajighasemi A., Hakimian R. (2015). Gender difference and root canal morphology in mandibular premolars: a cone-beam computed tomography study in an Iranian population. *Contemporary Clinical Dentistry*.

[23] Mashyakhy M., Gambarini G. (2019). Root and root canal morphology, differences between genders: a comprehensive in-vivo CBCT study in a Saudi population. *Acta Stomatol Croatica*.

[24] Martins J. N. R., Marques D., Leal Silva E. J. N., Caramês J., Mata A., Versiani M. A. (2020). Influence of demographic factors on the prevalence of a second root canal in mandibular anterior teeth-a systematic review and meta-analysis of cross-sectional studies using cone beam computed tomography. *Archives of Oral Biology*.

[25] Von Arx T. (2005). Frequency and type of canal isthmuses in first molars detected by endoscopic inspection during periradicular surgery. *International Endodontic Journal*.

[26] La S.-H., Jung D.-H., Kim E.-C., Min K.-S. (2010). Identification of independent middle mesial canal in mandibular first molar using cone-beam computed tomography imaging. *Journal of Endodontics*.

[27] Patel S., Dawood A., Ford T. P., Whaites E. (2007). The potential applications of cone beam computed tomography in the management of endodontic problems. *International Endodontic Journal*.

[28] Kuzekanani M., Walsh L. J., Haghani J., Kermani A. Z. (2017). Radix entomolaris in the mandibular molar teeth of an Iranian population. *International Journal of Dentistry*.

[29] Isola G., Polizzi A., Santonocito S., Alibrandi A., Ferlito S. (2019). Expression of salivary and serum malondialdehyde and lipid profile of patients with periodontitis and coronary heart disease. *International Journal of Molecular Sciences*.

[30] Isola G., Alibrandi A., Currò M. (2020). Evaluation of salivary and serum asymmetric dimethylarginine (ADMA) levels in patients with periodontal and cardiovascular disease as subclinical marker of cardiovascular risk. *Journal of Periodontology*.

[31] Isola G., Polizzi A., Iorio-Siciliano V., Alibrandi A., Ramaglia L., Leonardi R. (2020). Effectiveness of a nutraceutical agent in the non-surgical periodontal therapy: a randomized, controlled clinical trial. *Clinical Oral Investigations*.

